# Differentiation and Functional Comparison of Monocytes and Macrophages from hiPSCs with Peripheral Blood Derivatives

**DOI:** 10.1016/j.stemcr.2019.05.003

**Published:** 2019-06-11

**Authors:** Xu Cao, Gopala K. Yakala, Francijna E. van den Hil, Amy Cochrane, Christine L. Mummery, Valeria V. Orlova

**Affiliations:** 1 Department of Anatomy and Embryology, Leiden University Medical Center, Einthovenweg 20, 2333 ZC Leiden, the Netherlands

**Keywords:** hiPSC-derived monocytes, hiPSC-derived macrophages (IPSDMs), inflammation, monocyte adhesion under flow, efferocytosis, tumor phagocytosis

## Abstract

A renewable source of human monocytes and macrophages would be a valuable alternative to primary cells from peripheral blood (PB) in biomedical research. We developed an efficient protocol to derive monocytes and macrophages from human induced pluripotent stem cells (hiPSCs) and performed a functional comparison with PB-derived cells. hiPSC-derived monocytes were functional after cryopreservation and exhibited gene expression profiles comparable with PB-derived monocytes. Notably, hiPSC-derived monocytes were more activated with greater adhesion to endothelial cells under physiological flow. hiPSC-derived monocytes were successfully polarized to M1 and M2 macrophage subtypes, which showed similar pan- and subtype-specific gene and surface protein expression and cytokine secretion to PB-derived macrophages. hiPSC-derived macrophages exhibited higher endocytosis and efferocytosis and similar bacterial and tumor cell phagocytosis to PB-derived macrophages. In summary, we developed a robust protocol to generate hiPSC monocytes and macrophages from independent hiPSC lines that showed aspects of functional maturity comparable with those from PB.

## Highlights

• Robust derivation of monocytes and macrophages from multiple hiPSC lines
• Cryopreserved hiPSC monocytes are functional and adhere to endothelial cells
• hiPSC-derived macrophages are phenotypically similar to PB-derived macrophages
• hiPSC-derived macrophages can uptake AcLDL, bacteria, apoptotic cells, and tumor cells

## Teaser

In this article, Orlova and colleagues show that monocytes and macrophages differentiated from human induced pluripotent stem cells (hiPSCs) are functionally comparable with peripheral blood (PB)-derived cells, although some differences were identified: (1) hiPSC-derived monocytes were more activated and larger in size and (2) hiPSC-derived macrophages exhibited higher endocytosis and efferocytosis than PB-derived macrophages.

## Introduction

Human peripheral blood mononuclear cells (PBMCs) are routinely used to derive monocytes and macrophages for many areas of biomedical research, but despite the relative simplicity of the isolation procedure, it is often difficult outside specialized clinical centers to obtain large, high-quality cell batches on a regular basis from different donors, especially when research requires these from patients with rare diseases. In addition, recent studies suggest that many tissues are populated by specialist macrophages distinct from peripheral blood-derived macrophages (PBDMs) that are formed from primitive erythro-myeloid progenitors (EMPs) originating from hemogenic endothelium (HE) in the yolk sac (Ginhoux and Jung, 2014). Yolk sac-derived EMPs are different from hematopoietic stem cells (HSCs) derived from the aorta-gonad-mesonephros (AGM) region, which appears during the definitive stage of hematopoiesis and can be distinguished from AGM-derived hematopoietic progenitors by the absence of HOXA gene expression (Dou et al., 2016, Ivanovs et al., 2017, Ng et al., 2016). Studies using human pluripotent stem cells (hPSCs) showed that it is possible to differentiate yolk sac-like HE, identified as vascular endothelial cadherin (VEC)+, CD73−, and CD34+ cells, and early hematopoietic progenitors that express the hematopoietic marker CD43 (Choi et al., 2009, Choi et al., 2012). These CD43+ cells can give rise to EMP-like cells with broad erythroid and myeloid differentiation capacity, apparently reminiscent of EMPs found in the mouse embryo. Multiple protocols have shown that hPSCs could be a potent source of monocytes and macrophages (Choi et al., 2009, Happle et al., 2018, Karlsson et al., 2008, Lachmann et al., 2015, Lang et al., 2018, Schwartz et al., 2015, Takata et al., 2017, Uenishi et al., 2014, Vanhee et al., 2015, Zhang et al., 2015). Importantly, these hiPSC-derived macrophages (IPSDMs) are similar to yolk sac-derived EMPs, as they undergo MYB-independent myeloid differentiation (Buchrieser et al., 2017, Vanhee et al., 2015) and lack expression of HOXA genes (Dou et al., 2016, Ivanovs et al., 2017, Ng et al., 2016); this suggests they are more like tissue-resident macrophages than PBDMs. Furthermore, IPSDMs can be conditioned by the resident cells to acquire tissue-specific characteristics *in vitro* (Takata et al., 2017) and *in vivo* (Happle et al., 2018, Takata et al., 2017). hiPSCs, therefore, provide unique opportunities to study tissue-resident macrophages that are otherwise very difficult or impossible to access (Lee et al., 2018).

Previous protocols primarily utilized continuous harvesting of floating cells in culture over periods of up to 8 weeks, with average yields of 2- to 3 × 10^6^ IPSDMs per week per plate (Happle et al., 2018, Lachmann et al., 2015, van Wilgenburg et al., 2013). Continuous harvesting was recently successfully translated to stirred tank bioreactors for the mass production of IPSDMs (Ackermann et al., 2018). Here, we describe a protocol that allows production of EMP-like cells that can be further differentiated toward hiPSC-derived monocytes (hiPSC-mono) with a yield of 15 to 20 × 10^6^ from a single plate in just 15 days. These hiPSC-mono can be used immediately, or cryopreserved and used thereafter in functional assays or induced to differentiate into IPSDMs, and polarized to “classically activated” inflammatory (M1) or “alternatively activated” anti-inflammatory (M2) subtypes. We also performed a side-by-side comparison with PB-derived monocytes and macrophages using functional assays, including adhesion to endothelial cells (ECs) under flow, and phagocytosis of bacteria, apoptotic cells, and tumor cells.

## Results

### Differentiation of CD14+ Monocytes from hiPSCs

**Figure 1:**
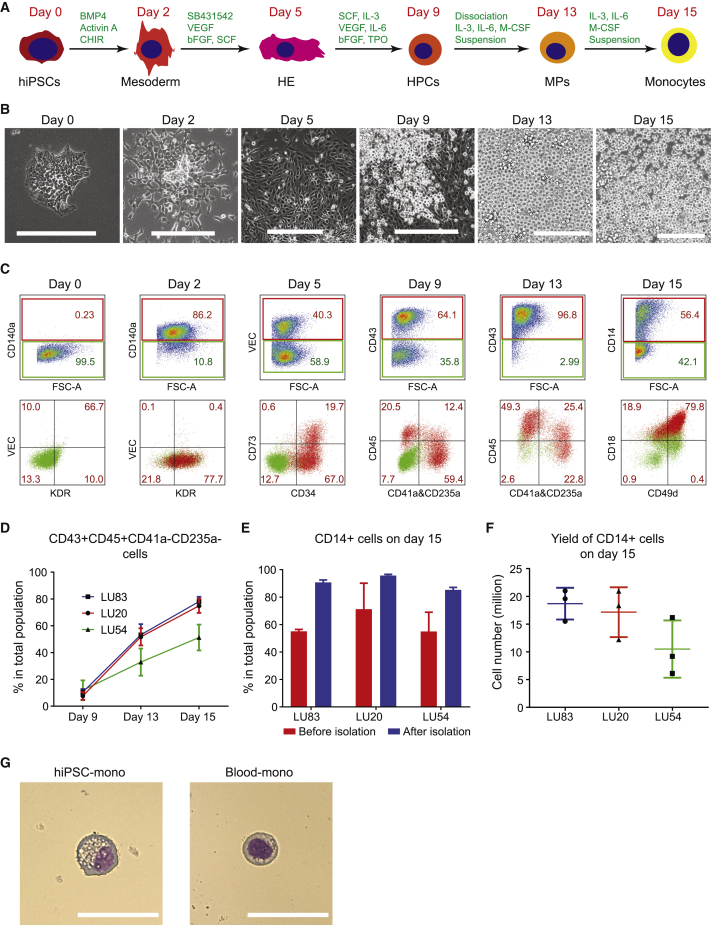
Differentiation of CD14+ Monocytes from hiPSCs (A) Schematic overview of CD14+ monocyte differentiation protocol from hiPSCs. (B) Bright-field images of representative cellular morphology at day 0 (undifferentiated hiPSCs), day 2 (mesoderm), day 5 (HE), day 9 (HPCs), day 13 (MPs), and day 15 (CD14+ monocytes). Scale bar represents 200 μm. (C) FACS analysis of stage-specific markers at day 0, day 2, day 5, day 9, day 13, and day 15 of differentiation from a representative hiPSC line (LU83). Positive populations are gated in the upper panels, and their percentages are shown in red in both upper and lower panels. (D) Quantification of the percentage of myeloid lineage cells (CD43+CD45+CD41a−CD235a−) in the total cell population at day 9, day 13, and day 15 of differentiation. Quantification of three independent experiments from three hiPSC lines (LU83, LU20, and LU54) is shown. (E) Quantification of the percentage of CD14+ cells at day 15 of differentiation before and after isolation using CD14+ MACS. Quantification of three independent experiments from three hiPSC lines (LU83, LU20, and LU54) is shown. (F) Yield of CD14+ monocytes at day 15 of differentiation from three hiPSC lines and three independent experiments. Yield of monocytes is equal to the total cell number multiplied by percentages of CD14+ cells. (G) Giemsa staining of hiPSC-mono isolated at day 15 of differentiation from one representative hiPSC line (LU83) and Blood-mono. Scale bar represents 50 μm. Error bars are ±SD of three independent experiments in (D–F). See also Figure S1 and Videos S1 and S2.

To derive monocytes from hiPSCs, we adapted our previous differentiation protocol of non-hemogenic VEC+CD73+ ECs (Orlova et al., 2014a, Orlova et al., 2014b) to conditions that allowed the derivation of VEC+ CD73− HE as described by Slukvin and colleagues (Uenishi et al., 2014). All differentiation steps were performed in IF9S serum-free medium (Uenishi et al., 2014), with some adaptations, such as normoxia (21% O_2_) and the timing of addition of growth factor. Undifferentiated hiPSCs were maintained in E8 medium and switched to IF9S medium. We found that 2 days of mesoderm induction with BMP4, Activin A, CHIR99021, followed by 3 days inducing endothelial cell fate with vascular endothelial growth factor (VEGF), SB431542, basic fibroblast growth factor (bFGF), and stem cell factor (SCF) resulted in efficient differentiation of VEC+CD73−CD34+ HE. From day 5, a combination of human recombinant interleukin (IL)-6, IL-3, thyroid peroxidase (TPO), SCF, FGF2, and VEGF was added to induce hematopoietic progenitor cells (HPCs) that resemble EMPs and were defined by the expression of CD43 and CD45. EMP-like cells were further differentiated into monocytes by addition of human macrophage colony-stimulating factor (M-CSF), IL-3, and IL-6 for another 6 days. The protocol was next tested in three independent hiPSC lines reprogrammed using non-integrating Sendai virus or episomal methods: LUMC0083 (LU83, from PB erythroblasts), LUMC0020 (LU20, from skin fibroblasts), and LUMC0054 (LU54, from kidney epithelial cells isolated from urine). After 2 days of mesoderm induction, more than 60% of cells were CD140a+ (Figures 1C, S1A, and S1B). On day 5, around 40% of the cells expressed endothelial cell-specific markers VEC and CD34. Within the VEC+CD34+ population, most cells were also CD73− HE (Figures 1C and S1A, and S1C). After another 4 days in the presence of hematopoietic growth factors and cytokines, many non-adherent HPCs emerged from adherent HE (Figure 1B and Videos S1 and S2). On day 9, expression of an early HPC surface marker CD43 was examined in the total population (adherent and suspension culture). Overall, by day 9, all three hiPSC lines had been induced to form HPCs expressing CD43 with high efficiency (Figures 1C, S1A, and S1D). At this stage, the majority of CD43+ cells were also CD41a+CD235a+, indicating they were erythro-megakaryocytic progenitors; only a small percentage of the total cell population being CD43+CD45+CD41a−CD235a− myeloid progenitors (Figures 1C, 1D, S1A, and S1E). The colony-forming unit (CFU) assay showed that cells on day 9 had already acquired high myeloid cell differentiation potential but had also developed the ability to differentiate into erythroid and granulocyte lineages (Figure S1F).

Video S1. Monocyte Differentiation Day 7 to Day 9, Related to Figure 1 Time-lapse imaging of monocyte differentiation from the LU83 hiPSC line. The video was taken from differentiation day 7 to day 9 in a time span of ∼50 h. The video is 15 frames/s. The interval between each frame is 40 min in real time. Scale bar represents 200 μm.

Video S2. Monocyte Differentiation Day 6 to Day 8, Related to Figure 1 Time-lapse imaging of monocyte differentiation from the LU83 hiPSC line. The video was taken from differentiation day 6 to day 8 in a time span of ∼48 h. The video is 15 frames/s. The interval between each frame is 16 min in real time. Scale bar represents 100 μm.

At day 9, cells were dissociated and cultured in suspension in the presence of IL-3 and IL-6 to promote proliferation of CD43+CD45+ myeloid progenitors and human M-CSF to promote differentiation of CD14+ monocytes from these myeloid progenitors (Choi et al., 2009, Uenishi et al., 2014). Under these conditions, the percentage of CD43+CD45+CD41a−CD235a− myeloid lineage cells increased to 50%–70% across the different hiPSC lines by day 15 (Figure 1D). Percentages of CD43+CD45−CD41a+CD235a+ erythro-megakaryocytic lineage cells rapidly decreased on day 13–15 (Figure S1E). By day 15, CD14+ cells represented more than half of the total population across three independent hiPSC lines (Figures 1C and S1A). CD14+ monocytes were then purified using immuno-magnetic beads (Figures 1E and S1G). Isolated CD14+ cells showed typical monocyte morphology (Figure 1G), although they were larger in size, with the cytoplasm containing fine and coarse vacuoles, most probably reflecting a more activated state (Figures S2A–S2C). After initially seeding 400,000 hiPSCs on a 12-well culture plate, 18.7 ± 2.9 million (LU83), 10.5 ± 5.2 million (LU20), and 17.2 ± 4.5 million (LU54) CD14+ monocytes, respectively, were harvested on day 15 (Figure 1F), resulting in a yield of 36.83 ± 10.40 monocytes generated from one hiPSC initially seeded or 15.47 ± 4.37 × 10^6^ (average of three lines) CD14+ monocytes from each 12-well plate. The isolated hiPSC-mono were cryopreserved for further functional assessment or differentiation into macrophages.

### Functional Assessment of hiPSC-Mono

**Figure 2:**
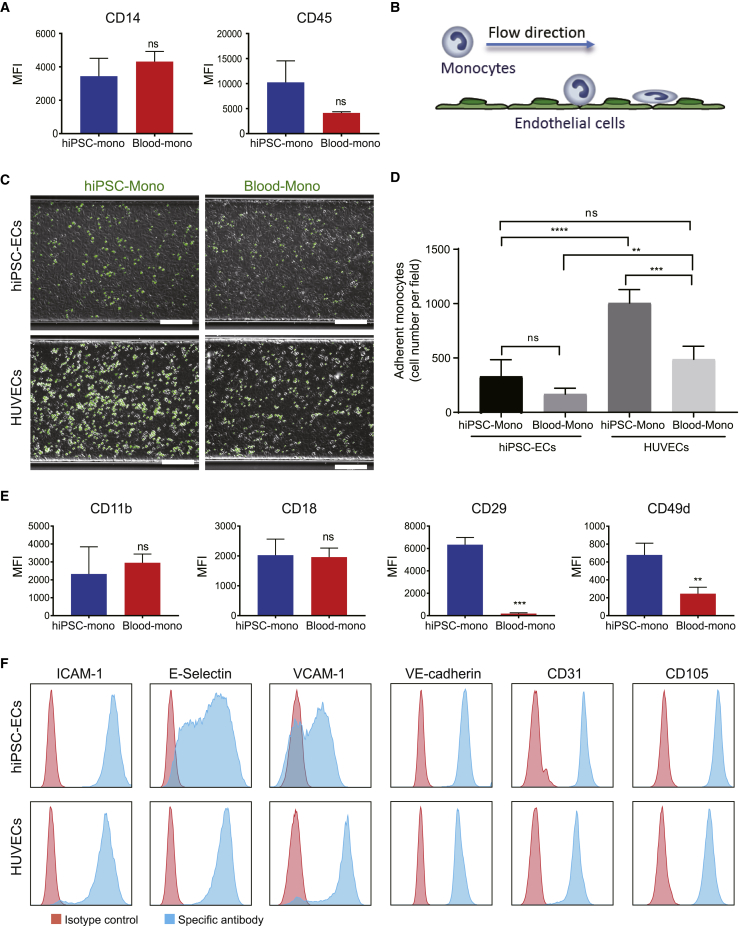
Functional Comparison of hiPSC-Mono and Blood-Mono in the Microfluidic Adhesion Assay (A) FACS analysis of surface expression of CD14 and CD45 on hiPSC-mono and Blood-mono after cryopreservation. Error bars are ±SD of three independent experiments. Unpaired t test: ns, non-significant. (B) Schematic for the microfluidic flow adhesion assay of monocytes and ECs. (C) Representative images taken at the end of the flow assay for each combination of ECs and monocytes. Monocytes were labeled with DiOC6 (green). Scale bar represents 200 μm. (D) Quantification of the number of adhered monocytes: hiPSC-mono and hiPSC-ECs, Blood-mono and hiPSC-ECs, hiPSC-mono and HUVECs, Blood-mono and HUVECs. Error bars are ±SD of four independent experiments. Uncorrected Fisher's least significant differences test: ns, non-significant; ^∗∗^p < 0.01, ^∗∗∗^p < 0.001, ^∗∗∗∗^p < 0.0001. (E) FACS analysis of surface expression of MAC-1 (CD11b and CD18) and VLA-4 (CD49d and CD29) integrin subunits on hiPSC-mono and Blood-mono. Error bars are ±SD of three independent experiments. Unpaired t test: ns, non-significant; ^∗∗^p < 0.01, ^∗∗∗^p < 0.001. (F) FACS analysis of ICAM-1, E-Selectin, VCAM-1, VE-cadherin, CD31, and CD105 on hiPSC-ECs and HUVECs after TNF-α treatment. Isotype control is shown in red and antigen-specific antibody is shown in blue. See also Figure S2.

Several cryopreserved batches of hiPSC-mono were thawed with a recovery of 43.2% ± 9.9% and were compared functionally with blood monocytes (Blood-mono) (Figure 2). Both cells expressed similar levels of monocyte surface markers CD14 and CD45 (Figure 2A). The monocytes were then compared functionally using a microfluidic monocyte adhesion assay to ECs we described previously (Halaidych et al., 2018) (Figure 2B). Briefly, hiPSC-mono or Blood-mono cells were inserted under flow into microfluidic chips coated with either primary human umbilical vein endothelial cells (HUVECs) or hiPSC-derived endothelial cells (hiPSC-ECs) stimulated with tumor necrosis factor alpha (TNF-α). Adhesion of the monocytes to ECs was determined under flow at venous shear stress (0.5 dyn/cm^2^). HUVECs expressed high levels of E-Selectin and VCAM-1 after TNF-α treatment compared with hiPSC-ECs, although they expressed comparable levels of ICAM-1 and endothelial cell-specific markers, such as VE-cadherin, CD31, and CD105 (Figure 2F). The total numbers of hiPSC-mono and Blood-mono adherent to HUVECs were higher than to hiPSC-ECs, as observed previously (Halaidych et al., 2018). On the other hand, the total number of hiPSC-mono adherent to ECs was higher than Blood-mono (Figures 2C and 2D). These differences correlated with increased expression of leukocyte integrin subunits CD49d and CD29 (VLA-4 integrin), the major ligands for the endothelial receptor VCAM-1, on hiPSC-mono compared with Blood-mono (Figure 2E).

### Differentiation of Macrophages from hiPSC-Mono

**Figure 3:**
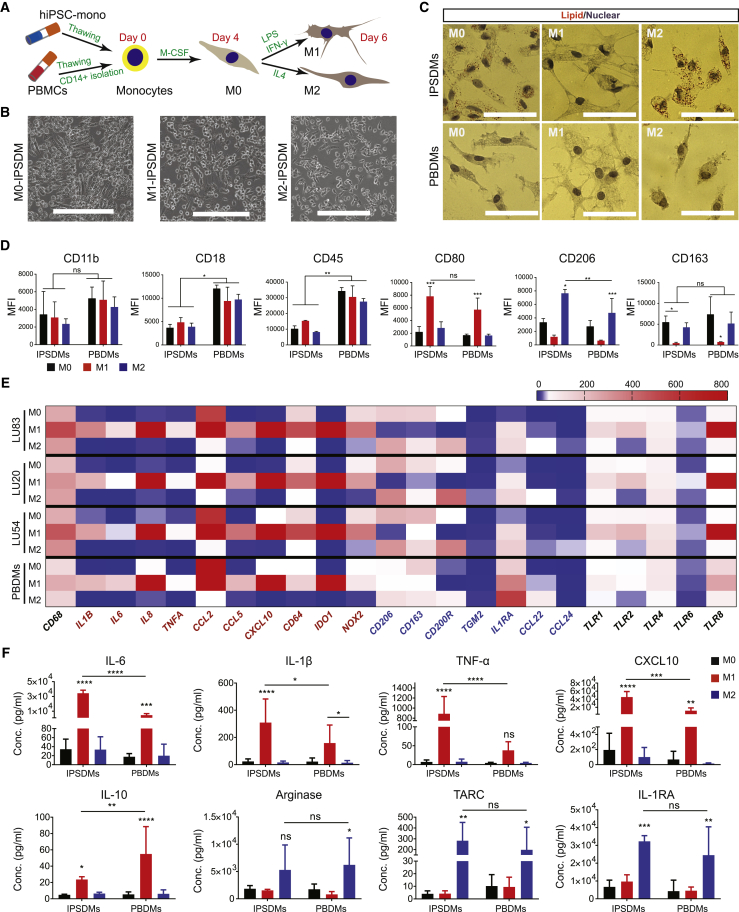
Characterization of IPSDMs (A) Schematic overview of the macrophage differentiation protocol from cryopreserved hiPSC-mono and PBMCs. (B) Bright-field images of representative cellular morphology of IPSDMs. Scale bar represents 200 μm. (C) Oil red O staining of lipid (red) within M0, M1, and M2 subtypes of IPSDMs and PBDMs. Nuclei (purple) were stained with hematoxylin. Scale bar represents 50 μm. (D) Quantification of surface expression of pan-specific macrophage markers, CD11b, CD18, and CD45, and subtype-specific markers, CD80 (M1) and CD206 (M2), on IPSDMs (differentiated from LU83) and PBDMs. Error bars are ±SD of three independent experiments. Uncorrected Fisher's least significant differences test: ns, non-significant; ^∗^p < 0.05, ^∗∗^p < 0.01, ^∗∗∗^p < 0.001. (E) Heatmap of gene expression analysis of macrophage-specific markers by qPCR in IPSDMs differentiated from three hiPSC lines (LU83, LU20, and LU54) and PBDMs. Mean values of three independent experiments are shown. M1-specific genes are shown in red and M2-specific genes are shown in blue. (F) Quantification of secreted cytokines and chemokines by a Multiplex assay using supernatants from IPSDMs and PBDMs after 48 h of polarization. Error bars are ±SD of three independent experiments. Uncorrected Fisher's least significant differences test: ns, non-significant; ^∗^p < 0.05, ^∗∗^p < 0.01, ^∗∗∗^p < 0.001, ^∗∗∗∗^p < 0.0001. See also Figure S3.

To differentiate toward M0 macrophages (M0), cryopreserved CD14+ hiPSC-mono or CD14+ Blood-mono isolated from cryopreserved PBMCs were plated on fetal calf serum (FCS)-coated cell culture plates in the presence of M-CSF for 4 days. The M0 cells could be polarized toward M1 macrophages (M1) using lipopolysaccharide (LPS) and interferon gamma (IFN-γ) or M2 macrophages (M2) using IL-4. The differentiation protocol is shown schematically in Figure 3A (Martinez and Gordon, 2014).

### Characterization of IPSDMs

Morphologically, IPSDMs were similar to PBDMs. Polarization toward M0 induced an elongated morphology. Polarization toward M1 resulted in a stellar shape with multiple protrusions, and polarization toward M2 resulted in a more rounded morphology (Figures 3B and 3C). Oil red O staining indicated that M0-IPSDMs and M2-IPSDMs had a higher intracellular lipid content compared with M1-IPSDMs and PBDMs (Figure 3C). Fluorescence-activated cell sorting (FACS) analysis of pan-specific macrophage surface markers CD11b, CD18, and CD45 showed comparable expression in all macrophage subtypes. IPSDMs expressed a comparable level of CD11b but a lower level of CD18 and CD45 compared with PBDMs. Subtype-specific macrophage markers CD80 (M1) and CD206 (M2) were highly expressed in the relevant IPSDM subsets and much like levels in PBDMs (Figures 3D, S3A, and S3B). M0-IPSDMs and M0-PBDMs also expressed high levels of M2 macrophage markers CD206 and CD163, in agreement with previous reports (Gordon and Martinez, 2010, Vogel et al., 2014) where M0 macrophages were indicated as already showing high similarity to M2 identity (Figures 3D, S3A, and S3B).

We next compared mRNA expression of macrophage pan-specific marker *CD68* and subset-specific markers (red text indicates M1 markers and blue text for M2 markers) in IPSDMs differentiated from three hiPSC lines to PBDMs. We also tested expression of toll-like receptors (TLRs), which are crucial for macrophage function, allowing the recognition of pathogens (Figure 3E). As expected, *CD68* was expressed by all macrophage subtypes. Expression of known pro-inflammatory cytokines and chemokines, including *IL1B*, *IL6*, *IL8*, *TNFA*, *CCL2*, *CCL5*, and *CXCL10* was the highest in the M1 subset of IPSDMs and PBDMs. Other known M1 markers, including *CD64*, *IDO1*, *NOX2,* were also highly expressed in M1-IPSDMs and M1-PBDMs. Gene expression of M2 markers, *CD206* and *CD163*, were indeed expressed highest in M2 subsets, and this matched well with surface protein levels (Figure 3D). M2-IPSDMs and M2-PBDMs subsets had the highest expression of M2-specific genes *CD200R* and *TGM2* and expressed the highest level of anti-inflammatory cytokines and chemokines, *IL1RA*, *CCL22*, and *CCL24*. TLRs, including *TLR1*, *TLR2*, *TLR4*, *TLR6*, and *TLR8* were preferentially expressed by M1, in accordance with previous work (Schlaepfer et al., 2014). Overall, the genes tested were comparable between IPSDMs and PBDMs (Figure 3E).

Cytokine and chemokine secretion were examined next in macrophage subtypes. The M1 subset of both IPSDMs and PBDMs secreted high levels of pro-inflammatory cytokines and chemokines, including IL-6, IL-1β, TNF-α, and CXCL10 (Figure 3F). The secretion of pro-inflammatory cytokines and chemokines was significantly higher in M1-IPSDMs than M1-PBDMs. Other pro-inflammatory cytokines and chemokines, including CCL2, IL-8, and IL-18, were also highly secreted by the M1 subset derived from different hiPSC lines and PBMC donors (Figure S3C). Notably, an anti-inflammatory cytokine, IL-10, was highly secreted by M1-IPSDMs and M1-PBDMs, in accordance with previous findings (de Waal Malefyt et al., 1991, Murthy et al., 2000, Stanley et al., 2012). The M2 subset of both IPSDMs and PBDMs secreted high levels of anti-inflammatory cytokines and enzymes such as TARC, IL-1RA, and Arginase (Figure 3F).

### Functional Characterization of IPSDMs

**Figure 4:**
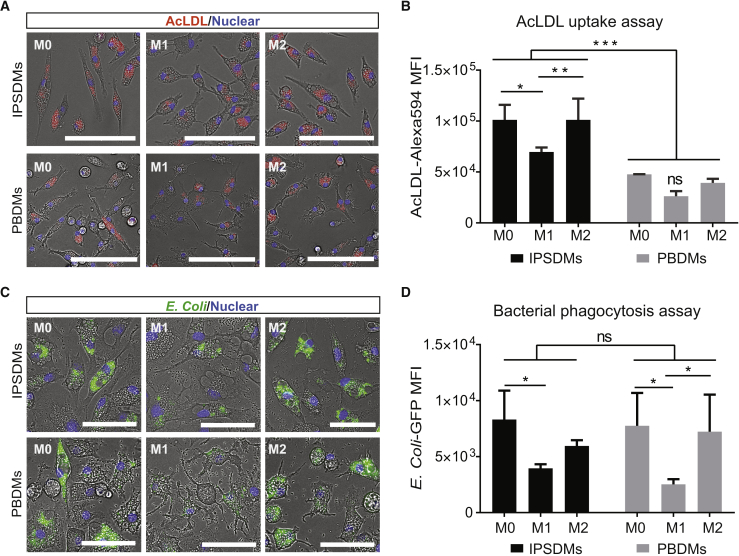
Endocytosis and Phagocytosis of Bacteria by IPSDMs and PBDMs (A) Representative images of the AcLDL-Alexa Fluor 594 uptake assay by different subtypes of IPSDMs and PBDMs. AcLDL positive uptake is shown in red; cell nuclei are stained with Hoechst in blue. Scale bar represents 100 μm. (B) Quantification of AcLDL-Alexa Fluor 594 median fluorescence intensity of different macrophage subtypes by FACS. Error bars are ±SD of three independent experiments. Uncorrected Fisher's least significant differences test: ns, non-significant; ^∗^p < 0.05, ^∗∗^p < 0.01, ^∗∗∗^p < 0.001. (C) Representative images of bacterial phagocytosis by different subtypes of IPSDMs and PBDMs. Nuclei were stained with Hoechst in blue. GFP-labeled (pHrodo green) *E. coli* were pH sensitive and only show green fluorescence inside macrophages. Scale bar represents 100 μm. (D) Quantification of *E. coli*-GFP median fluorescence intensities in macrophage subtypes by FACS. Error bars are ±SD of three independent experiments. Uncorrected Fisher's least significant differences test: ns, non-significant; ^∗^p < 0.05. IPSDMs were differentiated from LU83 in (A)–(D).

We next assessed the endocytic activity of IPSDMs using DiI-acetylated low-density lipoprotein (AcLDL) uptake. All IPSDM subtypes had the ability to ingest DiI-AcLDL. M1-IPSDMs showed significantly lower Dil-AcLDL uptake compared with M0-IPSDMs and M2-IPSDMs (Figures 4A and 4B). Moreover, compared with PBDMs, IPSDMs showed higher uptake of DiI-AcLDL (Figures 4A and 4B).

Next, we compared the ability of the IPSDMs to phagocytose bacteria. GFP-labeled *Escherichia coli* were incubated with IPSDMs and PBDMs, and their phagocytic efficiency was measured by FACS. M0-IPSDMs and M2-IPSDMs had the highest phagocytic activity compared with the pro-inflammatory M1-IPSDMs (Figures 4C and 4D). Furthermore, CD163, a crucial scavenger receptor mediating bacterial phagocytosis of macrophages, was mainly expressed by M0 and M2 macrophages and absent on M1, as shown in Figures 3D, 3E, S3A, and S3B. There was no significant difference between IPSDMs and PBDMs (Figures 4C and 4D).

### Assessment of Efferocytosis Activity of IPSDMs

**Figure 5:**
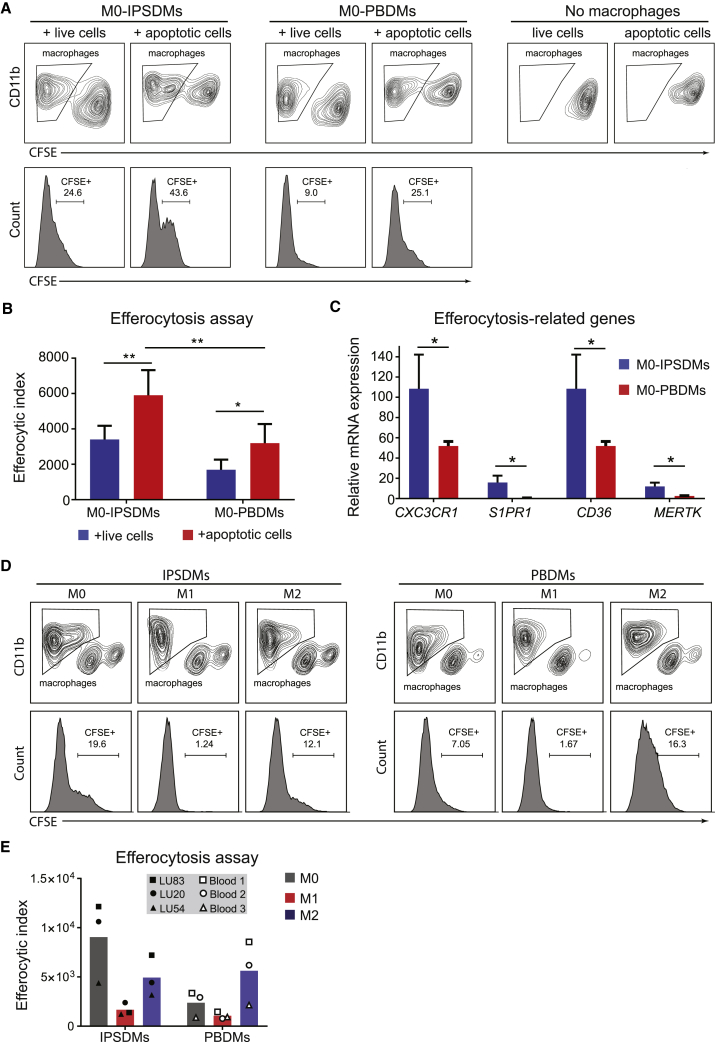
Characterization of Efferocytosis Activity of IPSDMs and PBDMs (A) Efferocytosis assay of M0-IPSDMs and M0-PBDMs. Live cells (used as a negative control) and apoptotic cells were labeled with CFSE, and macrophages were stained by anti-CD11b antibody. Histogram plots of CFSE (lower panel) within the CD11b+ population (upper panel) are shown. (B) Efferocytic index of M0-IPSDMs and M0-PBDMs. The percentage of CFSE+ macrophages was multiplied by the MFI of CFSE in order to calculate the efferocytic index. Error bars are ±SD of four independent experiments. Uncorrected Fisher's least significant differences test: ^∗^p < 0.05, ^∗∗^p < 0.01. (C) Quantification of gene expression of efferocytosis-related genes *CX3CR1*, *S1PR1*, *CD36*, and *MERTK* by qPCR in M0-IPSDMs and M0-PBDMs. Unpaired t test: ^∗^p < 0.05. (D) Efferocytosis assay of different subtypes of IPSDMs and PBDMs. Live cells (used as a negative control) and apoptotic cells were labeled with CFSE, and macrophages were stained by anti-CD11b antibody. Histogram plots of CFSE (lower panel) within the CD11b+ population (upper panel) are shown. (E) Efferocytic index of different subtypes of IPSDMs and PBDMs. The percentage of CFSE+ macrophages was multiplied by MFI of CFSE in order to calculate the efferocytic index. Data are presented as means of three biological replicates (three hiPSC lines or PBMC samples). IPSDMs were differentiated from LU83 in (A)–(D). See also Figure S4.

To determine whether IPSDMs can ingest apoptotic cells *in vitro*, we performed an efferocytosis assay. Apoptotic cells were obtained by exposing hiPSCs to UV radiation (35 J/cm^2^). More than half of the UV-treated cells became early apoptotic (Annexin V+propidium iodide [PI]−) with only 16.5% of the cells becoming necrotic (Annexin V+PI+) (Figure S4). Carboxyfluorescein succinimidyl ester (CFSE)-labeled apoptotic cells were then incubated with M0-IPSDMs and M0-PBDMs, and their efferocytosis efficiency was measured by FACS. Both M0-IPSDMs and M0-PBDMs showed higher efficiencies of apoptotic cell uptake than live cells without UV radiation. M0-IPSDMs showed higher efferocytosis activity than M0-PBDMs (Figures 5A and 5B). Receptors that mediate the “find-me” or “eat me” signals for efferocytosis, such as *CX3CR1*, *S1PR1*, *CD36*, and *MERTK*, were expressed at higher levels in M0-IPSDMs than M0-PBDMs (Figure 5C). Both M0- and M2-IPSDMs and PBDMs showed high efferocytosis capability, whereas M1-IPSDMs and M1-PBDMs showed poor efferocytosis (Figures 5D and 5E). This was confirmed across three independent hiPSC lines (Figure 5E).

### Assessment of Tumor Cell Phagocytotic Activity of IPSDMs

**Figure 6:**
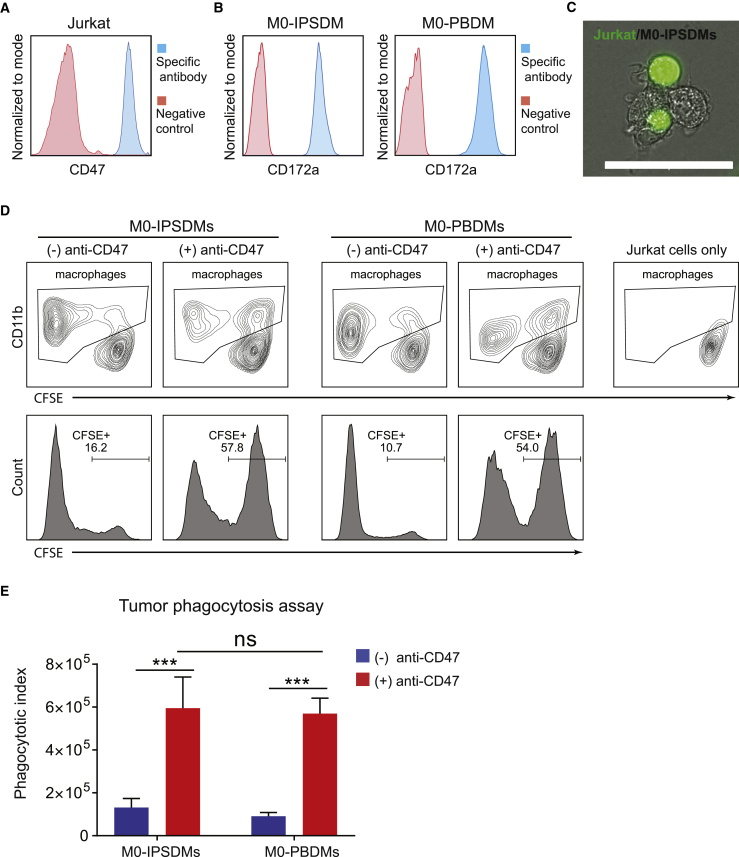
Phagocytosis of Tumor Cells by IPSDMs and PBDMs (A) FACS analysis of CD47 on Jurkat cells. Secondary antibody only was used as a negative control. (B) FACS analysis of CD172a on M0-IPSDMs and M0-PBDMs. Non-stained cells were used as negative control. (C) A representative image of Jurkat cells (labeled with green fluorescent dye) phagocytized by M0-IPSDMs (phase contrast image). Scale bar represents 50 μm. CFSE-labeled Jurkat cells were incubated with anti-CD47 blocking antibody and co-cultured with M0-IPSDMs for 30 min. (D) FACS analysis of Jurkat cell phagocytosis by M0-IPSDMs and M0-PBDMs. CFSE-labeled Jurkat cells were incubated with or without anti-CD47 blocking antibody and added to macrophages for 2 h. CD11b+ macrophages are gated (upper panel), and their CFSE intensity is shown as a histogram (lower panel). (E) Phagocytotic index of M0-IPSDMs and M0-PBDMs with and without CD47 blocking antibody. The percentage of CFSE+ macrophages was multiplied by the MFI of CFSE to get the phagocytotic index. Error bars are ±SD of four independent experiments. Uncorrected Fisher's least significant differences test: ns, non-significant; ^∗∗∗^p < 0.001. M0-IPSDMs were differentiated from LU83 in (B)–(E). See also Figure S5 and Video S3.

Previous studies demonstrated that macrophages show high infiltration in tumors; their ability to phagocytose tumor cells is currently being explored in cancer immunotherapy (Gul and van Egmond, 2015). CD47 overexpression on cancer cells often enables them to escape phagocytosis via the interaction with CD172a receptor on macrophages. This has led to the use of CD47 blocking antibody in multiple clinical trials to advance cancer therapy (Chao et al., 2012, Weiskopf and Weissman, 2015). Here, we compared the tumor cell phagocytosis ability of IPSDMs and PBDMs in the presence of a blocking CD47 antibody. Immortalized T cell lymphoma cells (Jurkat) were used as target tumor cells because they express high levels of CD47 (Figure 6A). M0-IPSDMs and M0-PBDMs expressed high and comparable levels of CD172a (Figure 6B). Pre-incubation with anti-CD47 antibody significantly increased engulfment of tumor cells by both IPSDMs (57.8%) and PBDMs (54.0%), compared with controls without CD47 blocking antibody (16.2% and 10.7%) (Figures 6C and 6D, Video S3, and data not shown). The phagocytic index (the product of CFSE mean fluorescence intensity [MFI] and percentage of CFSE+ macrophages) of IPSDMs and PBDMs increased around 5-fold and 7-fold, respectively, due to the CD47 block (Figure 6E). M0-IPSDMs showed similar tumor phagocytosis activity compared with M0-PBDMs in the presence of CD47-blocking antibody (Figures 6D and 6E). Tumor phagocytosis activity in the presence of anti-CD47 was next determined on M0-, M1-, and M2-IPSDMs, and we show that M0- and M2-IPSDMs had the highest tumor phagocytosis activity (Figures S5A and S5B).

Video S3. Tumor Phagocytosis by IPSDMs, Related to Figure 6 Tumor cell phagocytosis by M0-IPSDMs differentiated from LU83. The video was taken 30 min after co-culture of tumor cells with M0-IPSDMs. The video was made in the same field as Figure 6C. The video is 15 frames/s and the interval between each frame is 30 s in real time. Scale bar represents 50 μm.

## Discussion

PB-derived monocytes and macrophages have been widely used to study many diseases and tissue homeostasis. Recent studies, however, demonstrated that tissue-resident macrophages in mouse originate from yolk sac-derived EMPs that are different from PB or bone marrow-derived macrophages, which originate from HSCs. hPSCs could be efficiently differentiated toward EMP-like cells that are reminiscent of yolk sac-derived EMPs found in mouse, and represent MYB- and HOXA-independent lineages (Buchrieser et al., 2017, Dou et al., 2016, Ivanovs et al., 2017, Ng et al., 2016, Vanhee et al., 2015). Therefore, hPSC-derived monocytes and macrophages could potentially be a useful source of patient-specific cells that otherwise are difficult or impossible to derive. hPSC-derived monocytes and macrophages, therefore, can be used to study tissue-, organ-, and tumor-specific macrophages inaccessible through regular biopsies.

Here, we describe an efficient protocol that was robust over three independent hiPSC lines; derivative monocytes and macrophage subtypes were obtained with comparable differentiation efficiencies and functional properties. hiPSCs were first directed to generate mesoderm with high efficiency, then to form HE and HPCs from which monocyte-like cells could be derived after 15 days. As in earlier studies, cells emerging from the present protocol possibly resemble yolk sac-derived EMPs, although investigating HOXA expression or MYB dependence would be required to define their developmental identity more precisely. Magnetic bead-based purification of CD14+ cells allowed efficient enrichment to a >90% pure cell population, which could be cryopreserved, with a post-thaw recovery rate of 30%–50%. The additional magnetic bead-based purification step is the principal drawback of our protocol here compared with previously published protocols that involve continuous harvesting of macrophages (Ackermann et al., 2018, Buchrieser et al., 2017, Lachmann et al., 2015, van Wilgenburg et al., 2013, Zhang et al., 2015) and could limit the scalability of the protocol. Continuous harvesting protocols have clear advantages when large numbers of cells are needed, for example for transplantation studies. However, for disease modeling applications, a shorter protocol that allows cryopreservation of independent cell batches can have its own advantages. We found that the cell batches produced using our protocol are functionally indistinguishable, demonstrating the reproducibility of the production process.

Functional comparison with Blood-mono showed that hiPSC-mono apparently represent a more activated state, with cytoplasm containing fine and coarse vacuoles, greater aggregation in suspension culture, and increased surface expression of α4β1 integrin (VLA-4 or CD49dCD29). The activated phenotype of hiPSC-mono might either be due to prolonged culture or to differences in developmental origin. Using an *in vitro* model of inflammation in a microfluidic device, we found that the hiPSC-mono could roll and adhere to ECs much like primary Blood-mono. The hiPSC-mono could adhere to both HUVECs and hiPSC-ECs. The hiPSC-mono showed higher adherence to ECs than Blood-mono due to their higher surface expression of integrins CD49b and CD29, which gives them a greater affinity for EC surface receptors.

These hiPSC-mono could be differentiated toward macrophage lineages; the M0-IPSDMs could be polarized to form pro-inflammatory M1-IPSDMs or anti-inflammatory M2-IPSDMs. We confirmed the IPSDMs phenotype through their morphology, surface markers, mRNA expression, cytokine secretion, and responses in functional assays compared with PBDMs. M0-IPSDMs, M1-IPSDMs, and M2-IPSDMs all acquired typical morphologies and marker expression of the respective PBDM subtypes, as demonstrated previously using either fully defined or serum-based protocols (van Wilgenburg et al., 2013, Zhang et al., 2015). Importantly, M1-IPSDMs acquired a pro-inflammatory phenotype, and conversely, M0-IPSDMs and M2-IPSDMs showed an anti-inflammatory phenotype, based on secreted and surface markers and gene expression profiles. However, the endocytic activity of IPSDMs, determined by their ability to uptake AcLDL, was higher, and their ability to phagocytose apoptotic cells (or efferocytosis) was more efficient than PBDMs, much as demonstrated previously for tissue-resident macrophages (A-Gonzalez et al., 2017, Roberts et al., 2017). This has great potential for their use in the study of diseases such as autoimmune or cardiovascular diseases in which this mechanism is impaired.

Increasing evidence has brought macrophages to the fore in tumor immunotherapy. Like PBDMs, the IPSDMs here also expressed high levels of signal regulatory protein α (SIRPα, CD172a), the receptor for the “don't eat me” signal, CD47, which is highly expressed on tumor cells (Chao et al., 2012, Weiskopf and Weissman, 2015). We demonstrated that blocking CD172a−CD47 signaling in IPSDMs and PBDMs comparably increased tumor cell phagocytosis. This indicates that IPSDMs could be an alternative to PBDMs in developing new cancer immunotherapies.

In summary, we developed a highly robust protocol to derive monocytes from hiPSCs, which could be cryopreserved or differentiated toward M0-IPSDMs. These M0-IPSDMs could be further polarized to pro-inflammatory M1-IPSDMs and anti-inflammatory M2-IPSDMs. Again, these IPSDMs were phenotypically similar to PBDMs. The short differentiation time combined with serum-free medium, autologous source, high cell output, and reproducibility makes IPSDMs derived using this protocol an attractive cell source for disease modeling and provides a more consistent and reliable reference for downstream *in vivo* clinical trials.

## Experimental Procedures

### hiPSC Lines and Maintenance

The following hiPSC lines were used in the present study: LUMC0020 (LU20, generated from skin fibroblasts) (Zhang et al., 2014); LUMC0054 (LU54, generated from kidney epithelial cells isolated from urine, http://hpscreg.eu/cell-line/LUMCi001-A) (Halaidych et al., 2018); LUMC0083 (LU83, from PB erythroblasts). hiPSCs were cultured in recombinant vitronectin-coated plates in TeSR-E8, all from STEMCELL Technologies according to the manufacturer's instructions.

### Differentiation of Myeloid Cells from hiPSCs

hiPSCs were maintained in mTeSR-E8 to reach 80% confluence. On day −1, hiPSCs were dissociated with Gentle Cell Dissociation Reagent (STEMCELL Technologies) for 5 min at room temperature to obtain small cell clumps. The cells were then seeded into Matrigel-coated plates (75 μg/mL) at a density of ∼10,000 cell/cm^2^ (1:30 split ratio). Cells were cultured in TeSR-E8 for 24 h and switched to IF9S medium (Table S1), modified from Uenishi et al. (2014), supplemented with 50 ng/mL BMP4 (R&D Systems), 15 ng/mL ACTIVIN A (Miltenyi Biotec), and 1.5 μM CHIR99021 (Axon Medchem) for the first 2 days (day 0 to day 2). On day 2, cells were refreshed with IF9S supplemented with 50 ng/mL VEGF (R&D Systems), 50 ng/mL bFGF (PeproTech), 50 ng/mL SCF (Miltenyi Biotec), and 10 μM SB431542 (Tocris Bioscience). On day 5 and day 7, cells were refreshed with IF9S supplemented with 50 ng/mL VEGF, 50 ng/mL bFGF, 50 ng/mL SCF, 50 ng/mL IL-6 (Miltenyi Biotec), 50 ng/mL TPO (Miltenyi Biotec), and 10 ng/mL IL-3 (Miltenyi Biotec). On day 9, floating cells were collected, and adherent cells were dissociated with TrypLE (Life Technologies) for 10 min at 37°C. Then floating and adherent cells were combined and resuspended in IF9S medium supplemented with 50 ng/mL IL-6, 10 ng/mL IL-3, and 80 ng/mL M-CSF (Miltenyi Biotec). Cells collected from one 12-well plate were plated into one 24-well ultra-low attachment plate (Corning Life Sciences). Medium was refreshed on day 13 and day 15 with IF9S medium containing 50 ng/mL IL-6, 10 ng/mL IL-3, and 80 ng/mL M-CSF. Cells were cultured at 37°C, 5% CO_2_, under normoxia conditions throughout the differentiation.

### Isolation of CD14+ Myeloid Cells

On day 15 of differentiation, all cells in suspension were collected and washed once with FACS buffer (PBS, 0.5% BSA, 2 mM EDTA). Then, CD14+ cells were isolated using CD14 MicroBeads (Miltenyi Biotec) following the manufacturer's instructions; 60 μL of MicroBeads were used for 1 × 10^7^ total cells. Isolated CD14+ cells were cryopreserved in CryoStor CS10 medium (STEMCELL Technologies) or further differentiated into macrophages.

To isolate human Blood-mono, PBMCs were first isolated using Ficoll-Paque PLUS (GE Healthcare) from healthy donor blood. PBMCs were cryopreserved in CryoStor CS10 medium at a density of 20 million/mL. CD14+ monocytes were isolated from cryopreserved PBMCs using CD14 MicroBeads following the manufacturer's instructions.

### Cryopreservation of hiPSC-Mono

Isolated hiPSC-mono were centrifuged and suspended in CryoStor CS10 cryopreservation medium at a concentration of 3.75 × 10^6^ cells/mL, keeping the cell suspension on ice; 400 μL were aliquoted into each cryovial (1.5 × 10^6^ cells per vial). Cryovials were next placed in prechilled a Mr. Frosty Freezing Container and left at −80°C for 24 h, then transferred to liquid nitrogen for prolonged storage. To thaw hiPSC-mono, cryovials were removed from liquid nitrogen and thawed in a water bath at 37°C. hiPSC-mono were next transferred into 15 mL tubes containing 10 mL of prewarmed IF9S medium. Cells were centrifuged at 1,100 rpm for 3 min and suspended in IF9S medium supplemented with 80 ng/mL M-CSF. Cells were finally plated in FCS-coated cell culture plates and placed in the cell culture incubator for 48 h without disturbance.

### Differentiation of Macrophage Subtypes

hiPSC-derived CD14 cells or Blood-mono were plated on FCS-coated tissue culture plates at a density of 40,000 cells/cm^2^ in IF9S medium supplemented with 80 ng/mL M-CSF. After 4 days of culture, all monocytes differentiated into macrophages (M0) with more than 90% confluency. M0 macrophages were then polarized to M1 or M2 macrophages for 48 h in IF9S medium supplemented with different stimuli: 100 ng/mL LPS (Sigma) and 20 ng/mL IFN-γ (Miltenyi Biotec) for M1; 20 ng/m IL-4 (Miltenyi Biotec) for M2.

### CFU Assay

The hematopoietic CFU assay was performed using serum-free MethoCult SF H4636 (STEMCELL Technologies) following the manufacturer's instructions.

### Giemsa Staining

Monocytes were immobilized on microscope slides using Cytospin, followed by staining using Wright-Giemsa Stain (Sigma-Aldrich) according to the manufacturer's instructions.

### Differentiation of ECs from hiPSCs

hiPSCs were maintained in mTeSR-E8 and differentiated toward ECs using previously published protocols (Orlova et al., 2014a, Orlova et al., 2014b).

### Microfluidic Flow Assay

Microfluidic flow assay was performed as previously described (Halaidych et al., 2018). Briefly, Vena8 Endothelia+ chips (Cellix) were coated with 50 μg/mL fibronectin overnight at 4°C. ECs were first treated with 10 ng/mL BMP9 (R&D) for 24 h, then stimulated with TNF-α (10 ng/mL) for 12 h (overnight) in the presence of BMP9. Next day, ECs were collected and injected into the microfluidic channel. Then, the chip was incubated at 37°C to facilitate cell attachment. Monocytes were collected and stained with DiOC6 (1:5,000) (Sigma), then resuspended in IF9S medium at the end concentration of 2.5 × 10^6^ cells/mL. For flow experiments, monocytes were perfused for 5 min at 0.5 dyn/cm^2^ through the microfluidic channel, followed by a 5 min wash with IF9S medium. The number of adherent fluorescently labeled monocytes on ECs was quantified using the open source software CellProfiler (Carpenter et al., 2006).

### Oil Red O Staining

Macrophages were washed twice with DPBS and fixed with 4% paraformaldehyde for 15 min. Then, cells were washed three times with DPBS and stained with fresh Oil red O solution for 10 min followed by a wash with 75% ethanol for 15 s. After that, cells were stained with hematoxylin for 2 min and washed three times with DPBS.

### Flow Cytometry Analysis

Cells were washed once with FACS buffer and stained with antibodies for 30 min at 4°C. Samples were washed once with FACS buffer and analyzed on MACSQuant VYB (Miltenyi Biotech). The results were analyzed using Flowjo v10 (FlowJo, LLC). Fluorochrome conjugated human antibodies are listed in Table S2. FACS analysis of CD47 was done using anti-CD47 antibody (Bio-Rad, MCA911, 1:25) and Alexa 488 conjugated donkey anti-mouse secondary antibody (Thermo Fisher Scientific). PI solution (Miltenyi Biotec, 130-093-233, 1:100) was also used in specific flow cytometry analysis.

### Quantitative Real-Time Polymerase Chain Reaction

RNA was extracted from monocytes and macrophages using the NucleoSpin RNA kit (Macherey-Nagel). cDNA was synthesized using an iScript-cDNA Synthesis kit (Bio-Rad). iTaq Universal SYBR Green Supermixes (Bio-Rad) and Bio-Rad CFX384 real-time system were used for the PCR reaction and detection. Primers used are listed in Table S3. Relative gene expression was determined according to the standard delta Ct calculation and normalized to housekeeping genes (mean of hARP and RPL37A).

### Multiplex Cytokine Analysis

M0 macrophages were cultured in IF9S medium supplemented with 80 ng/mL M-CSF until reaching more than 90% confluence. Then, cells were polarized toward different subtypes of macrophages in IF9S medium containing the different stimuli indicated earlier. Cell culture supernatants were collected after 48 h of polarization. Concentration of cytokines was measured using a LEGENDplex Human Inflammation Panel kit and Human Macrophage/Microglia Panel kit (BioLegend) according to the manufacturer's instructions.

### AcLDL Uptake and Bacterial Phagocytosis Assay

M0 macrophages were dissociated and plated into 96-well plates (Corning Life Sciences) at a density of 50,000 cells/well in IF9S medium supplemented with 80 ng/mL M-CSF. After reaching more than 90% confluence, cells were polarized toward M0, M1, and M2 in IF9S medium for 12 h. Then cells were used for the AcLDL uptake or bacterial phagocytosis assay. Alexa Fluor 594 AcLDL (Thermo Fisher Scientific) was used for the AcLDL uptake assay following the manufacturer's instructions. The bacterial phagocytosis assay was done with pHrodo Green *E. coli* BioParticles Conjugate (Life Technologies) following the manufacturer's instructions. Finally, macrophages were dissociated with accutase (Promocell), and fluorescence intensities of the macrophages were measured by FACS using MACSQuant VYB.

### Efferocytosis Assay

M0 macrophages were dissociated and plated into 96-well plates at a density of 50,000 cells/well in IF9S medium supplemented with 80 ng/mL M-CSF. After reaching more than 90% confluence, cells were polarized toward M0, M1, and M2 in IF9S medium for 48 h. Then cells were ready for the efferocytosis assay. To obtain apoptotic cells, hiPSCs were dissociated and stained with 5 μM CFSE (Thermo Fisher Scientific) and exposed to 35 J/cm^2^ UV light for 5 min, then retained in medium for 90 min at 37°C. Then, 2 × 10^5^ apoptotic cells were added to each well of macrophages and incubated for 90 min at 37°C. Each well was washed once with IF9S medium to remove apoptotic cells that had not been phagocytosed. Then macrophages were dissociated with accutase (Promocell) and stained with CD11b antibody. Fluorescence intensity of CFSE in macrophages was measured by FACS. Percentages of CFSE+ cells within the CD11b+ population were multiplied by the CFSE MFI to calculate the efferocytic index.

### Tumor Phagocytosis Assay

M0 macrophages were plated into 96-well plates at a density of 50,000 cells/well and cultured in IF9S medium supplemented with 80 ng/mL M-CSF to reach more than 90% confluence. Jurkat tumor cells (kindly provided by Dr. Luuk Hawinkels, Leiden University Medical Center) were stained with CFSE and pre-incubated with 2 μg/mL anti-CD47 (Bio-Rad, MCA911) for 30 min. Then, 2 × 10^5^ Jurkat cells were added to each well of macrophages and incubated for 2 h at 37°C. Each well was washed once with IF9S medium, and macrophages were dissociated with accutase and stained with CD11b antibody. Fluorescence intensity of CFSE in macrophages was measured by FACS. The percentage of CFSE+ cells within the CD11b+ population was multiplied by the CFSE MFI to obtain the phagocytic index.

### Statistical Analysis

Statistical analysis was conducted with GraphPad Prism 7 software. Two-way ANOVA with uncorrected Fisher's least significant differences test was applied for the analysis of two independent variables. Comparison between two samples was done with the unpaired t test. More details are described in the figure legends. Error bars are shown as mean ± SD. ns, non-significant, ^∗^p < 0.05, ^∗∗^p < 0.01, ^∗∗∗^p < 0.001, ^∗∗∗∗^p < 0.0001.

## Author Contributions

X.C. and G.K.Y. designed and performed the research, analyzed and the interpreted results, and wrote the manuscript; F.E.vdH performed real-time PCRs; A.C. analyzed and interpreted the results and wrote the manuscript; C.L.M. designed the research and edited the manuscript; V.V.O. designed the research, analyzed and interpreted results, and wrote the manuscript.

## Supplemental Information

Document S1. Figures S1–S5 and Tables S1–S3

Document S2. Article plus Supplemental Information
