# Supplementary material for: Differentiation and Functional Comparison of Monocytes and Macrophages from hiPSCs with Peripheral Blood Derivatives
Source: Stem Cell Reports. 2019 Jun 11;12(6):1282–97. doi: 10.1016/j.stemcr.2019.05.003 (PMC6565887; doi:10.1016/j.stemcr.2019.05.003)
Supplement: Document S1. Figures S1–S5 and Tables S1–S3 [file mmc1.pdf]

Stem Cell Reports, Volume *12*

Supplemental Information

Differentiation and Functional Comparison of Monocytes and Macrophages from hiPSCs with Peripheral Blood Derivatives

Xu Cao, Gopala K. Yakala, Francijna E. van den Hil, Amy Cochrane, Christine L. Mummery, and Valeria V. Orlova

Inventory of Supplemental Information

Supplemental Figures and Legends:

Figure S1. Differentiation of CD14+ monocytes from hiPSCs. Related to Figure 1.
Figure S2. Comparison of cell sizes of whole blood, PBMCs, Blood-mono and hiPSC-mono. Related to Figure 2.
Figure S3. Characterization of IPSDMs and PBDMs. Related to Figure 3.
Figure S4. Induction of Apoptosis by UV Radiation. Related to Figure 5.
Figure S5. Characterization IPSDMs tumor phagocytosis activity. Related to Figure 6.

Supplemental Tables

Table S1. Formulation for IF9S medium. Related to Experiment Procedures.
Table S2. List of conjugated antibodies. Related to Experiment Procedures.
Table S3. Sequence of primes used for qPCR. Related to Experiment Procedures.

Supplemental Videos

Movie S1. Monocyte differentiation day 7 to day 9. Related to Figure 1.
Movie S2. Monocyte differentiation day 6 to day 8. Related to Figure 1.
Movie S3. Tumor phagocytosis by IPSDMs. Related to Figure 6.

Supplemental Figures and Legend

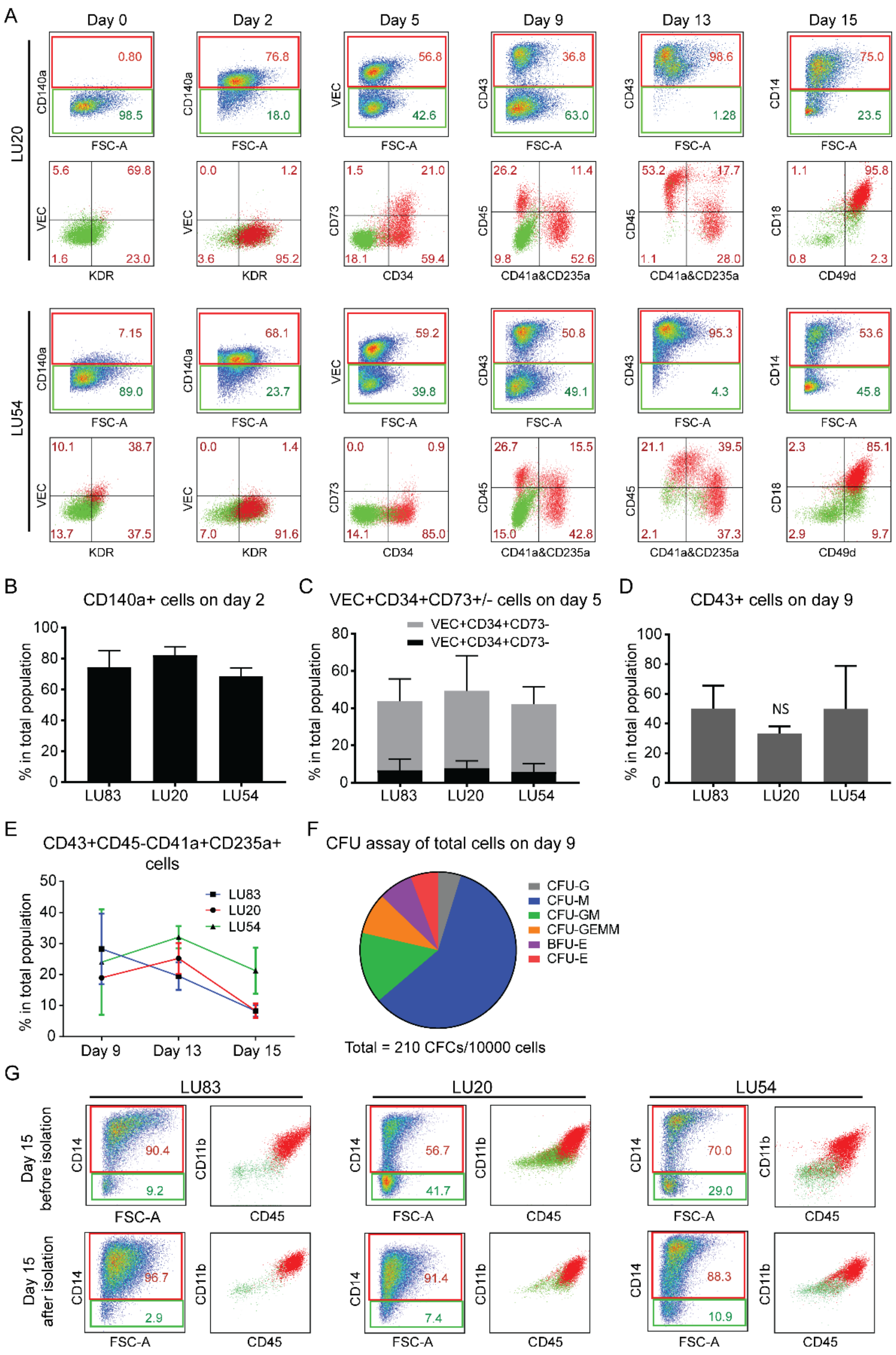

(Figure legend see next page)

Figure S1. Differentiation of CD14+ monocytes from hiPSCs. Related to Figure 1.
(A) FACS analysis of stage-specific markers at day 0, day 2, day 5, day 9, day 13 and day 15 of differentiation from LU20 and LU54. Positive populations are gated in upper panels and their percentages are shown in red in both upper and lower panels. (B) Percentage of early pan-mesodermal cell marker (PDGFRα) on day 2 of differentiation from three hiPSC lines (LU83, LU20 and LU54). (C) Percentage of non-HE (VEC+CD34+CD73+) and HE (VEC+CD34+CD73-) subsets on day 5 of differentiation from three hiPSC lines (LU83, LU20 and LU54). (D) Percentage of early HPC marker CD43 on day 9 of differentiation from three hiPSC lines (LU83, LU20 and LU54). (E) Percentage of erythro-megakaryocytic lineage cells (CD43+CD45-CD41a+CD235a+) in total cell population on day 9, day 13 and day 15 of differentiation from three hiPSC lines (LU83, LU20 and LU54). (F) CFU assay of total cell population on day 9 of differentiation from LU83. (G) Representative FACS analysis of CD14+ monocytes before and after MACS isolation on day 15 of differentiation from three hiPSC lines (LU83, LU20 and LU54). Error bars are ±SD of three independent experiments in (B-E).

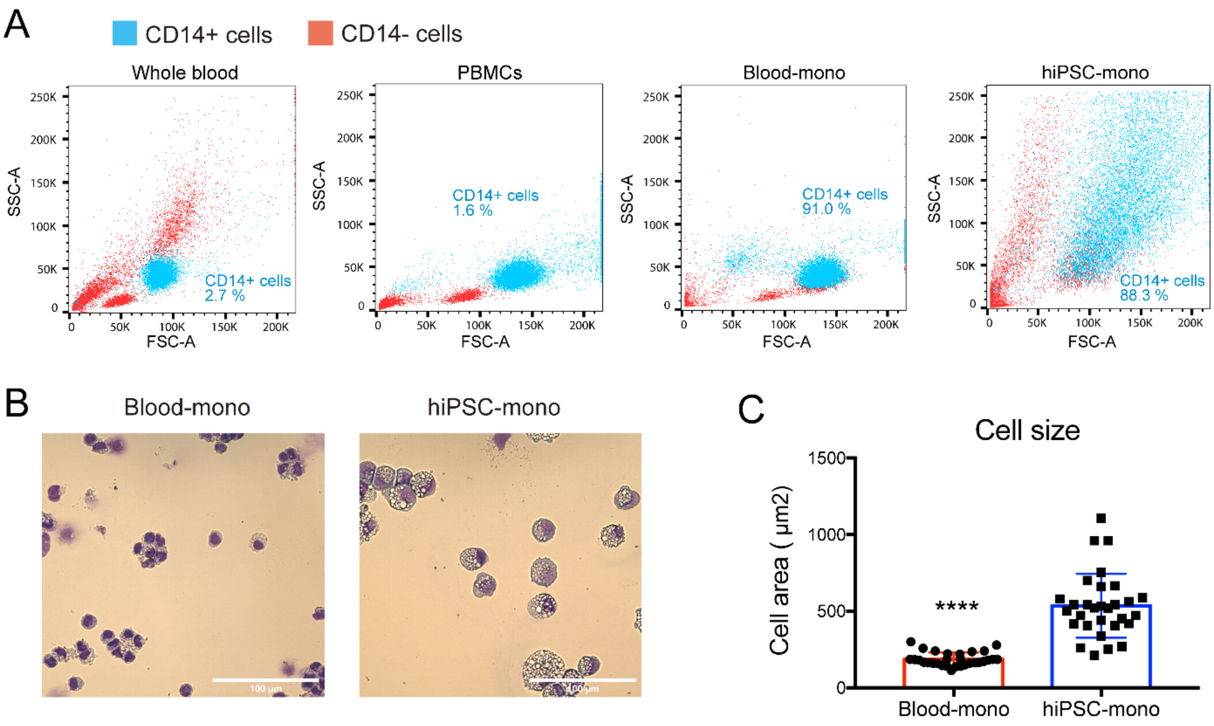

Figure S2. Comparison of cell sizes of whole blood, PBMCs, Blood-mono and hiPSC-mono. Related to Figure 2.

(A) FACS analysis of whole blood, PBMCs, Blood-mono from the same donor and hiPSC-mono on day 15 of differentiation from LU83 hiPSC line. (B) Giemsa staining of blood-mono isolated from human PBMC and hiPSC-mono isolated on differentiation day 15. Scale bar 100 µm. (C) Quantification of cell size of blood-mono and hiPSC-mono using Giemsa staining images. Cell area of 30 intact cells was measured from each cell type. Unpaired t-test. ****$p < 0.0001$.

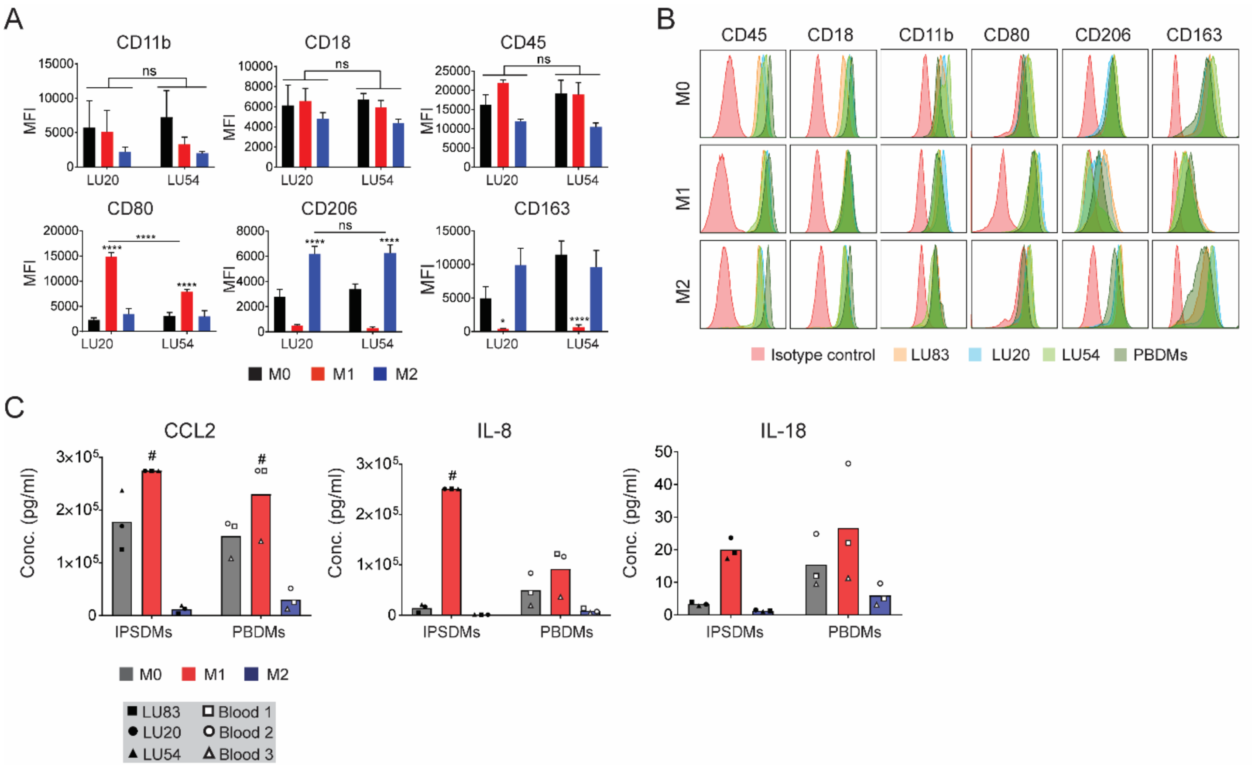

Figure S3. Characterization of IPSDMs and PBDMs. Related to Figure 3.
(A) Quantification of surface expression of pan-specific macrophage markers: CD11b, CD18 and CD45 and subtype-specific markers: CD80 (M1) and CD206 and CD163 (M2) on IPSDMs (differentiated from LU20 and LU54). Error bars are ±SD of three independent experiments. Uncorrected Fisher's LSD test. ns = non-significant, $*p < 0.05$, $****p < 0.0001$. (B) Representative FACS plots of pan-specific macrophage markers: CD11b, CD18 and CD45 and subtype-specific markers: CD80 (M1), CD206 and CD163 (M2) on IPSDMs (differentiated from LU20 and LU54) and PBDMs. (C) Quantification of secreted cytokines and chemokines by Multiplex assay using supernatants from IPSDMs and PBDMs after 48hours of polarization. Data are presented as mean of three biological replicates (three hiPSC lines or PBMC samples). # higher than the detection limit of Multiplex.

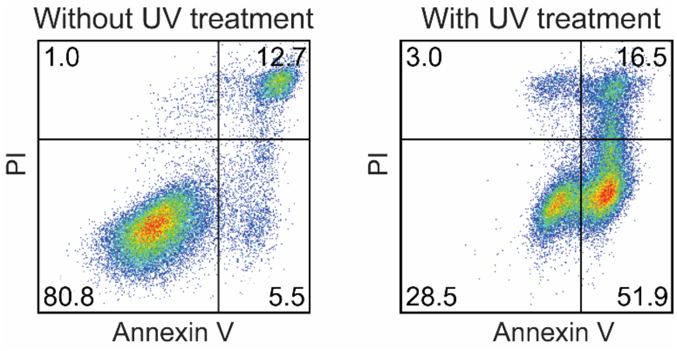

Figure S4. Induction of Apoptosis by UV Radiation. Related to Figure 5.
FACS analysis of apoptotic (Annexin V+ PI-) cells in hiPSCs without and with UV (35 J/cm2) treatment.

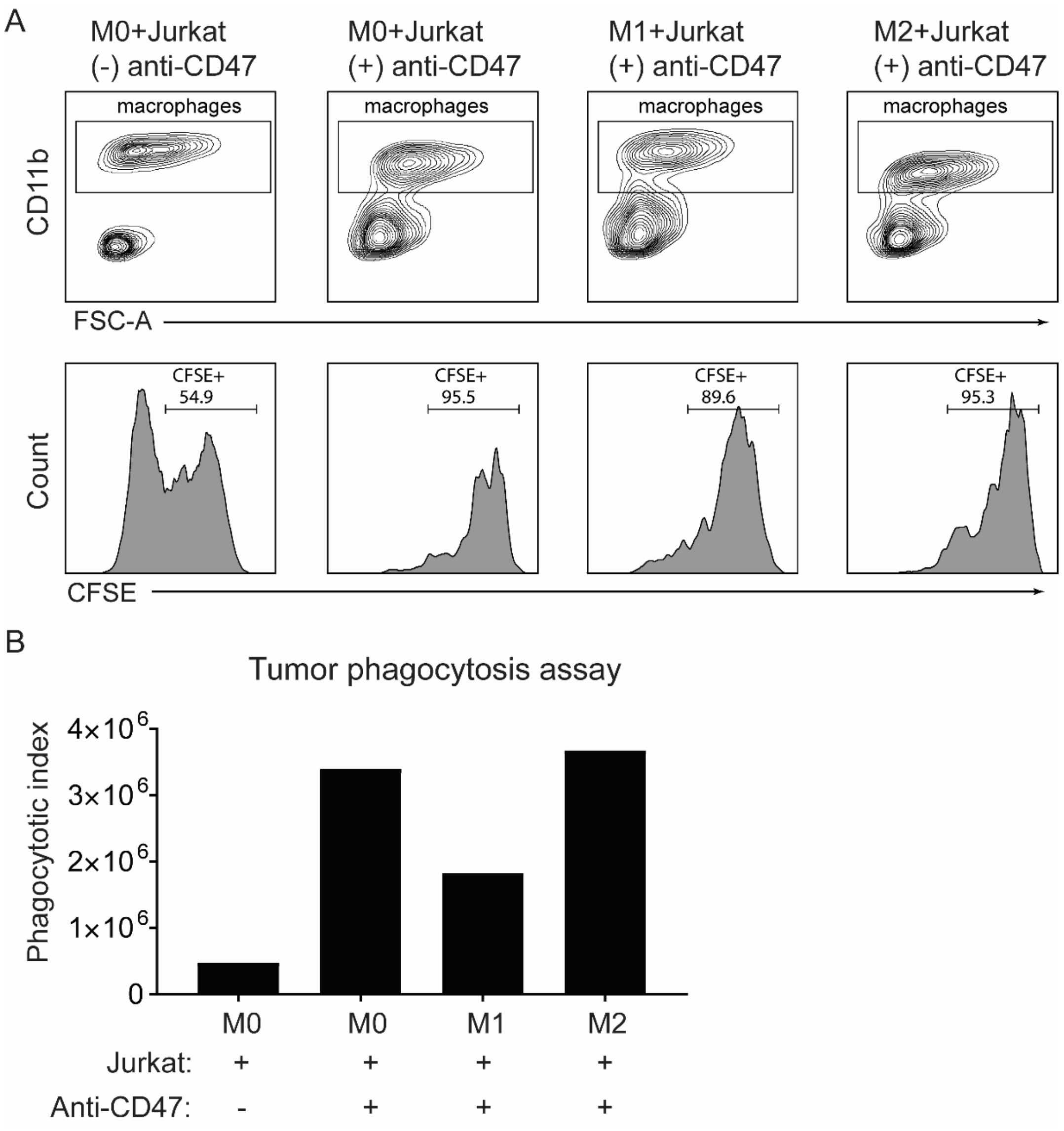

Figure S5. Characterization IPSDMs tumour phagocytosis activity. Related to Figure 6.
(A) FACS analysis of Jurkat cell phagocytosis by different subtypes of IPSDMs in the presence of CD47 blocking antibody. Jurkat cell phagocytosis by IPSDMs (M0) without CD47 blocking antibody is shown as a negative control. CD11b+ IPSDMs are gated (upper panel) and their CFSE intensities are shown as a histogram (lower panel). (B) Phagocytotic index of different subtypes of IPSDMs in the presence of CD47 blocking antibody. Jurkat cell phagocytosis by IPSDMs (M0) without CD47 blocking antibody is shown as a negative control. Percentage of CFSE+ macrophages was multiplied by MFI of CFSE to obtain the phagocytotic index. IPSDMs were differentiated from LU83 in (A-B).

Supplemental Tables

Medium component (stock concentration)	Source	Volume added (250ml final volume)	Final concentration
**IMDM**	Iscove's modified Dulbecco's medium (IMDM), no phenol red (Gibco, cat. no. 21056-023)	117.25 ml	--
**F12**	Ham's F-12 nutrient mix, GlutaMAX supplement (Gibco, cat. no. 31765-027)	117.25 ml	--
**PVA (5%)**	Poly vinyl alcohol (Sigma-Aldrich, cat. no. P8136-250G)	50 ul	10 mg/L
**Lipids (100X)**	Chemically defined lipid concentrate (Gibco, cat. no. 11905031)	250ul	0.1% (vol%)
**ITS-X (100X)**	Insulin-transferrin-selenium-ethanolamine (Gibco, cat. no. 51500-056)	5 ml	2% (vol%)
**αMTG (1.3% in IMDM)**	Mono-thio glycerol (Sigma-Aldrich, cat. no. M6145-25ml)	750 µl	40 ul/L
**AA2P (5 mg/ml)**	Sigma-Aldrich, cat. no. A8960	3.2 ml	64 mg/L
**GlutaMax (100X)**	GlutaMAX-1 supplement (Gibco, cat. no. 35050-038)	2.5 ml	1% (vol%)
**NEAA (100X)**	MEM Non-Essential Amino Acids Solution (100X) （Gibico, Cat. No. 11140-035）	2.5 ml	1% (vol%)
**Pen-strep (5,000 U/ml)**	Gibco, cat no. 15070-063	1.25ml	0.5% (vol%)

Table S1. Formulation for IF9S medium. Related to Experiment Procedures.

Antibody	Fluorochrome	Source	Dilution	Catalog #
**CD140a**	BV421	BD Bioscience	1:100	562799
**VE-Cadherin**	Alexa488	eBioscience	1:50	53-1449-42
**CD34**	APC	Miltenyi Biotec	1:20	130-090-954
**KDR**	PE	R&D	1:20	FAB357P
**CD73**	PE	BD Pharmingen	1:20	550257
**CD43**	PE	BD Bioscience	1:20	560199
**CD45**	FITC	Miltenyi Biotec	1:20	130-080-202
**CD41a**	Vioblue	Miltenyi Biotec	1:20	130-105-610
**CD235a**	Vioblue	Miltenyi Biotec	1:20	130-100-273
**CD14**	PE	Miltenyi Biotec	1:20	130-091-242
**CD11b**	Vioblue	Miltenyi Biotec	1:20	130-097-336
**CD18**	FITC	Miltenyi Biotec	1:20	130-101-237
**CD49d**	PE-Vio770	Miltenyi Biotec	1:20	130-104-326
**CD29**	PE	eBioscience	1:50	12-0299-71
**ICAM1**	F	R&D	1:20	BBA20
**E-Selectin**	F	R&D	1:20	BBA21
**VCAM1**	PE	R&D	1:20	FAB5649P
**CD31**	APC	eBioscience	1:50	17-0319
**CD105**	Vioblue	Miltenyi Biotec	1:20	130-099-666
**CD80**	PE-Vio770	Miltenyi Biotec	1:20	130-101-218
**CD206**	FITC	Miltenyi Biotec	1:20	130-095-131
**CD163**	FITC	Miltenyi Biotec	1:100	130-112-290
**CD172a**	PE-Vio770	Miltenyi Biotec	1:20	130-099-793
**Annexin-V**	Pacific Blue	Thermofisher	1:20	A35122

Table S2. List of conjugated antibodies. Related to Experiment Procedures.

Gene	Forward sequence	Reverse sequence	Product size
CD68	GGAAATGCCACGGTTCATCCA	TGGGGTTCAGTACAGAGATGC	247
IL1B	ATGATGGCTTATTACAGTGGCAA	GTCGGAGATTCGTAGCTGGA	132
IL6	ACTCACCTCTTCAGAACGAATTG	CCATCTTTGGAAGGTTCAGGTTG	149
IL8	AGCACTCCTTGGCAAAACTG	CGGAAGGAACCATCTCACTG	116
TNFA	CCTCTCTCTAATCAGCCCTCTG	GAGGACCTGGGAGTAGATGAG	220
CCL2	CAGCCAGATGCAATCAATGCC	TGGAATCCTGAACCCACTTCT	190
CCL5	CCAGCAGTCGTCTTTGTCAC	CTCTGGGTTGGCACACACTT	54
CXCL10	GTGGCATTCAAGGAGTACCTC	TGATGGCCTTCGATTCTGGATT	198
CD64	AGCTGTGAAACAAAGTTGCTCT	GGTCTTGCTGCCCATGTAGA	75
IDO1	GCCAGCTTCGAGAAAGAGTTG	ATCCCAGAACTAGACGTGCAA	96
NOX2	ACCGGGTTTATGATATTCCACCT	GATTTCGACAGACTGGCAAGA	135
CD206	TCCGGGTGCTGTTCTCCTA	CCAGTCTGTTTTTGATGGCACT	211
CD163	TTTGTCAACTTGAGTCCCTTCAC	TCCCGCTACACTTGTTTTCAC	127
CD200R	TGGTTGTTGAAAGTCAATGGCT	CTCAGATGCCTTCACCTTGTTT	153
TGM2	GAGGAGCTGGTCTTAGAGAGG	CGGTCACGACACTGAAGGTG	184
IL1RA	CATTGAGCCTCATGCTCTGTT	CGCTGTCTGAGCGGATGAA	167
CCL22	ATCGCCTACAGACTGCACTC	GACGGTAACGGACGTAATCAC	129
CCL24	ACATCATCCCTACGGGCTCT	CTTGGGGTCGCCACAGAAC	176
TLR1	CCACGTTCCTAAAGACCTATCCC	CCAAGTGCTTGAGGTTCACAG	248
TLR2	ATCCTCCAATCAGGCTTCTCT	GGACAGGTCAAGGCTTTTTACA	118
TLR4	AGACCTGTCCCTGAACCCTAT	CGATGGACTTCTAAACCAGCCA	147
TLR6	TTCTCCGACGGAAATGAATTTGC	CAGCGGTAGGTCTTTTGGAAC	75
TLR8	ATGTTCCTTCAGTCGTCAATGC	TTGCTGCACTCTGCAATAACT	143
CX3CR1	ACTTTGAGTACGATGATTTGGCT	GGTAAATGTCGGTGACACTCTT	177
S1PR1	TTCCACCGACCCATGTACTAT	GCGAGGAGACTGAACACGG	185
CD36	GGCTGTGACCGGAACTGTG	AGGTCTCCAACTGGCATTAGAA	92
MERTK	CTCTGGCGTAGAGCTATCACT	AGGCTGGGTTGGTGAAAACA	162
RPL37A	GTGGTTCCTGCATGAAGACAGTG	TTCTGATGGCGGACTTTACCG	84
HARP	CACCATTGAAATCCTGAGTGATGT	TGACCAGCCCAAAGGAGAAG	116

Table S3. Sequence of primes used for qPCR. Related to Experiment Procedures.

Supplemental Videos

Movie S1. Monocyte differentiation day 7 to day 9. Related to Figure 1.
Time-lapse imaging of monocyte differentiation from LU83 hiPSC line. Video was taken from differentiation day 7 to day 9 in a timespan of ~50 hours. The video is 15 frames/second. The interval between each frame is 40 minutes in a real time. Scale bar represents 200 μm.

Movie S2. Monocyte differentiation day 6 to day 8. Related to Figure 1.
Time-lapse imaging of monocyte differentiation from LU83 hiPSC line. Video was taken from differentiation day 6 to day 8 in a timespan of ~48 hours. The video is 15 frames/second. The interval between each frame is 16 minutes in a real time. Scale bar represents 100 μm.

Movie S3. Tumour phagocytosis by IPSDMs. Related to Figure 6.
Tumor cell phagocytosis by M0-IPSDMs differentiated from LU83. Video was taken 30 minutes after co-culture of tumor cells with M0-IPSDMs. Video was made in the same field as Figure 6C. Video is 15 frames/second and interval between each frame is 30 seconds in a real time. Scale bar represents 50 μm.
